# *Toxoplasma gondii* infection in sheep from Romania

**DOI:** 10.1186/s13071-022-05634-8

**Published:** 2023-01-23

**Authors:** Anamaria Ioana Paștiu, Viorica Mircean, Aurélien Mercier, Karine Passebosc-Faure, Nicolas Plault, Marie-Laure Dardé, Radu Blaga, Isabelle Villena, Dana Liana Pusta, Anamaria Cozma-Petruț, Adriana Györke

**Affiliations:** 1 Department of Parasitology and Parasitic Diseases, Faculty of Veterinary Medicine, University of Agricultural Sciences and Veterinary Medicine Cluj‐Napoca, 3‐5 Calea Mănăştur Street, 400372 Cluj‐Napoca, Cluj-Napoca Romania; 2 Department of Genetics and Hereditary Diseases, Faculty of Veterinary Medicine, University of Agricultural Sciences and Veterinary Medicine Cluj‐Napoca, 3‐5 Calea Mănăştur Street, 400372 Cluj‐Napoca, Cluj-Napoca Romania; 3 Inserm U1094, IRD U270, Univ. Limoges, CHU Limoges, EpiMaCT - Epidémiologie des maladies chroniques en zone tropicale, Institut d’Epidémiologie et de Neurologie Tropicale, OmegaHealth, Limoges, France; 4 Centre National de Référence Toxoplasmose, CHU Limoges, 2 Martin Luther King Street, 87042 Limoges, France; 5 Anses, INRAE, Ecole Nationale Vétérinaire d’Alfort, Laboratoire de Santé Animale, BIPAR, 94700 Maisons-Alfort, France; 6 EA 7510 ESCAPE, SFR CAP-SANTE, University of Reims Champagne-Ardenne, Reims, France; 7 National Reference Centre On Toxoplasmosis/Toxoplasma Biological Resource Center, CHU Reims, General Koening Street, Reims, France; 8 Department of Bromatology, Hygiene, Nutrition, Faculty of Pharmacy, “Iuliu Haţieganu” University of Medicine and Pharmacy, 6 Pasteur Street, 400349 Cluj-Napoca, Cluj-Napoca Romania

**Keywords:** *Toxoplasma gondii*, Sheep, Lambs, Serology, Bioassay, PCR, Microsatellite genotyping

## Abstract

**Background:**

Toxoplasmosis is a widespread zoonosis caused by the intracellular protozoan parasite *Toxoplasma gondii.* Limited epidemiological information is available about the prevalence of *T. gondii* in sheep in Romania, and a high incidence would have implications for both the economy and public health. To our knowledge, no studies are available about the *T. gondii* strains circulating in lambs. The objective of this study was to assess the prevalence of *T. gondii* in sheep (serology), lambs (serology, bioassay, PCR) and sheep abortions (PCR) in Romania. Moreover, the study aimed to perform the genetic characterization of *T. gondii* isolates from lambs.

**Methods:**

Serum samples collected from 2650 sheep (2067 adults and 583 lambs) were tested for anti-*T. gondii* antibodies (IgG) using a commercial ELISA kit. Likewise, 328 pairs of diaphragmatic muscle-serum samples were collected from lambs aged between 2 and 4 months. Lamb serum samples were analyzed using MAT for anti-*T. gondii* antibody detection. The diaphragm tissue samples from MAT-positive lambs (at a dilution ≥ 1:25) were bioassayed in mice. The *T. gondii* strains were genotyped using 15 microsatellites markers. Additionally, brain and heart samples from 76 sheep abortions were analyzed for *T. gondii* DNA by polymerase chain reaction (PCR) targeting the 529-bp repeat region (REP529).

**Results:**

The results showed that more than half of the tested sheep were *T. gondii* seropositive (53.5%). The seroprevalence was significantly higher in adults (61.1%) than in lambs (26.4%). The seroprevalence of *T. gondii* infection in slaughtered lambs, by MAT, was 37.5% (123/328). There were bioassayed in mice 56 diaphragmatic tissues from 123 seropositive lambs. *Toxoplasma gondii* strains were isolated from 18 (32.1%) lambs intended for human consumption. All *T. gondii* strains were confirmed by PCR. Six strains were genotyped using 15 microsatellite markers and belonged to genotype II. *Toxoplasma gondii* DNA was detected in 11.8% (9/76) of sheep abortions.

**Conclusions:**

The present study showed the presence of *T. gondii* in sheep in all the regions considered in the study. The high prevalence of *T. gondii* infection in sheep and lambs, demonstrated by serology, molecular analysis and bioassay, highlighted that there is an important risk of human infection in consuming raw or undercooked sheep/lamb meat.

**Graphical Abstract:**

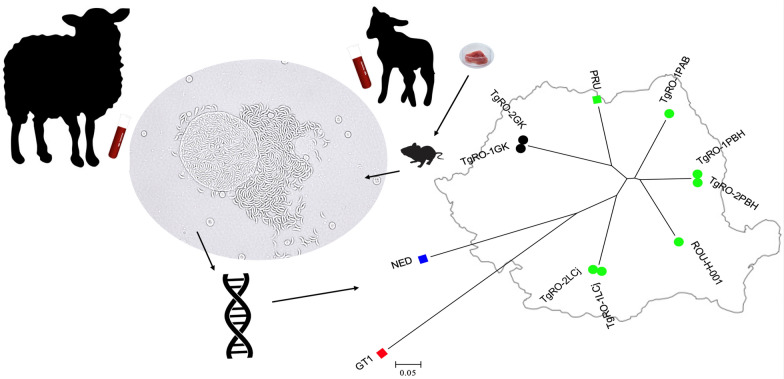

## Background

*Toxoplasma gondii,* Phylum Apicomplexa, is a zoonotic parasite with worldwide distribution. The parasite has a heteroxenous life cycle with *Felidae* as definitive host (asexual and sexual stages in the intestinal epithelium) and warm-blooded animals, including humans, as intermediate hosts (asexual extraintestinal stage, mainly in the brain and muscles) [1]. The infective stages are represented by the sporozoites from sporulated oocysts, the bradyzoites from tissue cysts and the tachyzoites [2]. Human infections can be acquired by the ingestion of sporulated oocysts from water or vegetables and tissue cysts from raw or undercooked meat. Also, the congenital transmission of tachyzoites represents an important route of infection for the fetus.

Toxoplasmosis in sheep was first described by Hartley et al. [3] and Hartley and Marshall [4]. Although cats and wild felids are considered the predominant source of infection in sheep due to continuous contamination of pastures with *T. gondii* oocysts, congenital transmission should not be forgotten [5]. In general, most sheep become *T. gondii* seropositive after birth, < 2% are infected congenitally and < 4% of the positive ewes transmit the parasite transplacentally [6]. After infection with *T. gondii*, sheep develop humoral and cellular-mediated immune responses against the parasite, which provides effective protection against the parasite during subsequent pregnancies [6]. However, *T. gondii* is a major cause of abortion and stillbirth in sheep worldwide, causing significant economic losses to breeders due to infertility, reproduction disorders, loss of offspring and reduction of milk production [7, 8].

Furthermore, the infection of sheep with *T. gondii* can have important implications for public health. In countries with a temperate climate, between 30 and 63% of *T. gondii* infections in humans have been attributed to the consumption of raw or undercooked meat and/or meat products [9]. Sheep meat is recognized worldwide as a major source of toxoplasmosis [10]. A positive correlation has been demonstrated between the consumption of sheep meat and the prevalence of toxoplasmosis in humans [11]. The ingestion of raw or insufficiently heat-processed meat contaminated with *T. gondii* may facilitate zoonotic transmission of the parasite, which can be present in almost all edible sheep tissues [12]. A recent study showed an uneven distribution of *T. gondii* cysts in muscle samples from lambs and indicated that even small amounts of meat (5–10 g) can have the potential to transmit *T. gondii* [13].

Besides congenital transmission, *T. gondii* can cause severe clinical symptoms in individuals with a deficient immune system, such as HIV/AIDS patients, oncology patients or transplant recipients. For these immunocompromised individuals, recent studies have demonstrated an increased risk of acquiring *T. gondii* infection [14].

In Romania, sheep breeding was one of the oldest occupations, and the phenomenon that defines the history of shepherding is transhumance. A country with a great tradition of and experience in sheep breeding, in 2020 Romania ranked third in the European Union, after Spain and Greece, in terms of small ruminants herds, with 11,189 million head [15]. The sheep are raised mainly for milk and meat production. Lamb meat is consumed especially during the Easter holidays.

Seroprevalence of anti-*T. gondii* antibodies in humans in northwestern and central Romania was 59.5%, higher in rural (63.6%) than urban areas (55.1%) [16]. The annual risk of infection is estimated to be 3–4.5% for children/adolescences, 1–2% for adults and 5.5% for people aged up to 65 years old [16].

Considering the epidemiological situation of *T. gondii* in humans in our country, the economic importance of sheep toxoplasmosis, as well as the limited amount of data regarding the toxoplasmosis in sheep in our country, the purpose of this study was to estimate the prevalence of *T. gondii* infection in sheep and lambs in Romania. The study was divided in four substudies: (1) seroepidemiological study; (2) bioassay study; (3) study on *T. gondii* prevalence in aborted fetuses; (4) the genetic diversity of the isolated strains from lambs.

## Methods

### Seroepidemiological study

**Fig. 1:**
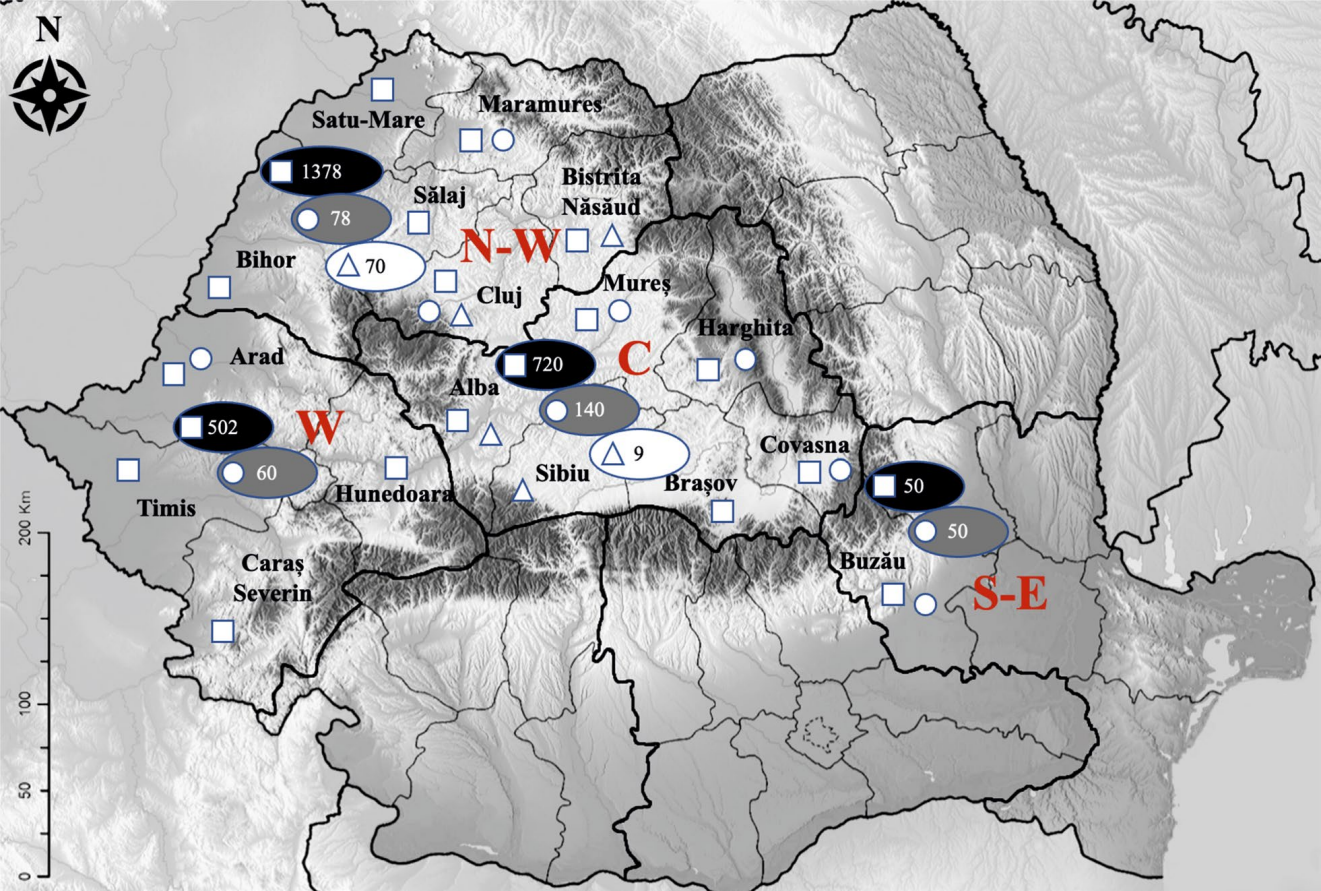
Regional distribution of sheep included in the study in the counties and regions in Romania. The squares indicate the counties of origin of sheep for serological study; the circles indicate the counties of origin of lambs for *T. gondii* isolation study; triangles indicate counties of origin of sheep abortions. The total number of sheep per region for serological study is indicated by the black ovals. The total number of lambs per region for *T. gondii* isolation study is indicated by the gray ovals. The total number of sheep abortions per region is indicated by the white ovals

A total of 2650 blood samples were collected by puncture of the jugular vein or during slaughtering of randomly selected adult sheep (*n* = 2067; age = 1–6 years old) and lambs (*n* = 583; age = 2–8 months old). The sheep originated in 16 counties from four regions of Romania: central (Alba, Mureș, Brașov, Harghita, Covasna), northwestern (Bihor, Cluj, Sălaj, Satu-Mare, Maramureș, Bistrița-Năsăud), western (Arad, Timiș, Caraș-Severin, Hunedoara) and southeast (Buzău) (Fig. 1). The sheep were reared in herds with 50–1000 animals per unit. The sheep herds were allowed to graze from spring to autumn, and spring waters represented the water main source. The samples were collected with the consent of sheep breeders or of the abattoir veterinarian. The sheep breeders voluntarily participated in the study. Data regarding the age, county and region were collected (Table 1, Fig. 1). The samples were refrigerated and transported on the same day to the laboratory. The blood samples were centrifuged at 3000 rpm for 10 min, and the sera were stored at − 20 °C until further analysis.

**Table 1:** Seroprevalence of *T. gondii* infection in adult sheep and lambs in Romania by ELISA

Region	Total				Adults ≥ 1 year old				Lambs < 1 year old			
	*n*	Positive (*n*)	Prevalence (95% CI)	*P* value	*n*	Positive (*n*)	Prevalence (95% CI)	*P* value	*n*	Positive (*n*)	Prevalence (95% CI)	*P* value
Central	720	290	40.28% (36.76–43.90)	0.001	421	235	55.82% (51.04–60.49)	0.001	299	55	18.39% (14.17–23.26)	0.001
Northwest	1378	774	56.17% (53.53–58.77)		1251	732	58.51% (55.76–61.21)		127	42	33.07% (24.98–41.97)	
West	502	348	69.32 (65.15–73.20)*		395	296	74.94% (70.44–78.96)*		107	52	48.6 (38.3–58.5)*	
Southeast	50	5	10 (3.33–21.81)		NA	NA	NA		50	5	10 (3.3–21.8)	
Total	2650	1417	53.47 (51.57–55.36)		2067	1263	61.1 (58.98–63.18)		583	154	26.4 (22.9–30.14)	

*P < 0.05; *n* = number of samples; CI = confidence interval; NA = not applicable

*Toxoplasma gondii*-specific IgG antibodies were detected by enzyme-linked immunosorbent assay (ELISA) using a commercial kit (Chekit Toxotest Antibody ELISA, Idexx-Bommeli, Switzerland) following the manufacturers’ instructions. The test results were interpreted as positive, doubtful or negative.

### Bioassay study

During slaughtering, paired samples of diaphragmatic muscle (5–25 g) and blood were collected randomly from 328 lambs from seven counties from four regions of Romania: central (Mureș, Harghita, Covasna), northwestern (Cluj, Maramureș), western (Arad) and southeast (Buzău) (Fig. 1). The lambs were 2–4 months old and weighed 8–25 kg. They were slaughtered during the Easter holiday and intended for familial consumption. A questionnaire was prepared for collection of data regarding the age and region of origin of the animals (Table 3). The samples were transported under refrigerated conditions to the laboratory. Serum samples were tested by modified agglutination test (MAT) within 24–48 h. Diaphragmatic tissue samples were kept under refrigerated conditions (4 °C) until bioassay in mice was performed.

The MAT for the detection of *T. gondii*-specific IgG antibodies was performed as previously described [17], using an antigen prepared from formalin-fixed whole RH strain tachyzoites (Reims, France). Each serum sample was serially twofold diluted. The threshold dilution was 1:6, and dilutions continued until the end of seropositivity. All serum samples with a titer ≥ 1:6 were considered positive.

The diaphragm tissue samples from lambs identified as seropositive by MAT at a dilution ≥ 1:25 were bioassayed on pathogen-free Swiss mice. The protocol described in the study of Paştiu et al. [18] was used. In brief, each diaphragm sample was minced and mixed (Knife Mill Grindomix GM 200, Retsch, Haan, Germany) with digestion solution (0.25% trypsin (93,613, Sigma-Aldrich, Saint Louis, MO, USA)/0.025% EDTA). The homogenate was incubated at 37 °C for 1.5 h and then filtered through gauze. The suspension was centrifuged at 1800×*g* for 10 min, the supernatant was discarded, and the pellet (digest) was washed with PBS, pH 7.2 (phosphate-buffered saline), by centrifugation three times. The digest was mixed with 3 ml PBS containing 100 μl antibiotic solution (20,000 units penicillin/10,000 units streptomycin, P0781, Sigma-Aldrich) [19]. For each sample, 0.5 ml of digest was inoculated intraperitoneally in two Swiss mice. Twice daily, the mice were monitored. After 4 weeks, all the surviving mice were killed and bled, and the sera were tested by MAT for anti-*T. gondii* IgG-specific antibody detection. After death of the mice, their brains were recovered. If signs of acute toxoplasmosis (ruffled fur, diminished response to handling, state of prostration, severe weight loss and neurological signs) were observed for 3 consecutive days; the mice had been killed earlier. Mouse brains were mechanically homogenized and examined microscopically for brain cyst search and counting. For each brain, five glass slides were prepared. Also, the mouse brains were analyzed by conventional PCR (cPCR) targeting 529-bp repeat element (RE) [20]. The isolated strains of *T. gondii* were subsequently genotyped.

### Toxoplasma gondii prevalence in aborted fetuses

A total of 76 aborted sheep fetuses (*n* = 67) and dead born lambs (*n* = 9) were collected and analyzed for the presence of *T. gondii*. Aborted fetuses, originated from two counties in central Romania (Alba, Sibiu) and two counties in northwestern Romania (Cluj, Bistriţa-Năsăud) (Fig. 1), were brought by the sheep breeders for investigations at the Faculty of Veterinary Medicine, Cluj-Napoca. The aborted fetuses were expelled at different periods of gestation. Samples of heart (*n* = 76) and brain (*n* = 73) were collected from each aborted fetus. The samples were kept at – 20 °C until processing by cPCR [20].

### Polymerase chain reaction (PCR)

DNA was extracted from mouse brains and aborted fetus tissues (heart and brain) using a commercial kit (Isolate Genomic II DNA Kit, Meridian Bioscience, USA) according to the manufacturer’s protocol. Positive and negative controls were represented by DNA obtained from *T. gondii* RH strain and tissue from free-pathogen Swiss mice, respectively. *Toxoplasma gondii* DNA was amplified by conventional PCR using the pair primers Tox 4 (5′ CGC TGC AGG GAG GAA GAC GAA AGT TG 3′) and Tox 5 (5′ CGC TGC AGA CAC AGT GCA TCT GGA TT 3′) that amplify the 529-bp RE (REP529, GenBank accession no. AF146527) as previously described [20]. Positive and negative controls and distilled ultrapure water were included in each run.

### Genotyping analysis

DNA samples extracted from brain cysts in mice positive for *T. gondii* DNA were submitted to genotyping using 15 microsatellite markers (N61, B18, M33, M48, TUB2, N83, XI.1, N82, TgM-A, W35, IV.1, B17, N60, M102, AA). In brief, for each primer pair, the forward primer was 5′-end labeled with fluorescein to allow sizing of PCR products electrophoresed in an automatic sequencer: 6-carboxyfluorescein (6-FAM) was used for TUB2, XI.1, B18, N83, N61, M33 and M48; hexachlorofluorescein (HEX) for MS TgM-A, B17, N82, W35 and IV.1; and 2,7′,8′-benzo-5′-fluoro-2′,4,7-trichloro- 5-carboxyfluorescein (NED) for AA, N60 and M102. PCR was carried out in a 25-µl reaction mixture consisting of 12.5 µl 2x QIAGEN Multiplex PCR Master Mix (Qiagen, Courtaboeuf, France), 5 pmol of each primer and 5 µl DNA. After an initial 15 min denaturation at 95 °C, 35 cycles were performed as follows: 30 s at 94 °C, 3 min at 61 °C, 30 s at 72 °C. This was followed by a final extension for 30 min at 60 °C. PCR products were diluted 1:10 with deionized formamide. One microliter of each diluted PCR product was mixed with 0.5 µl of a dye-labeled size standard (ROX 500, Applied Biosystems, Life Technologies, Carlsbad, CA) and 23.5 µl deionized formamide (Applied Biosystems). This mixture was denatured at 95 °C for 5 min and then electrophoresed using an automatic sequencer (ABI PRISM 3130xl, Applied Biosystems). The sizes of the alleles in bp were estimated using GeneMapper analysis software (version 5.0, Applied Biosystems).

The microsatellite data were used for producing an unrooted neighbor-joining tree using Populations 1.2.32 (http://bioinformatics.org/populations/) based on Cavalli-Sforza and Edwards [21] chord distance estimator and generated with MEGA7 (http://www.megasoftware.net/history.php).

### Statistical analysis

Point estimates and 95% confidence intervals (95% CI) for the anti-*T. gondii* antibodies and *T. gondii* DNA were calculated. These parameters were determined overall and by age group (lambs < 1 year; adults ≥ 1 year) and for each region (central, northwest, west, southeast). Also, the estimated age of lambs was recorded (between 2 and 8 months), and six groups were formed (2 months; 3 months; 4 months; 5 months; 6 months; 8 months). The difference in prevalence among groups was statistically analyzed using a Chi-square test of independence. A *P*-value of < 0.05 was considered statistically significant. Data were processed using EpiInfo 2000 software (CDC, Atlanta, GA, USA) (http://wwwn.cdc.gov/epiinfo).

## Results

### Seroepidemiological study

The overall seroprevalence of *T. gondii* antibodies in sheep by ELISA was 53.5% (1417/2650; 95% CI: 51.57–55.36%). Furthermore, 4.6% (121/2650; 95% CI: 3.8–5.4%) of the sera obtained doubtful results (Table 1).

The seroprevalence was significantly higher in adult sheep (61.10%; 1263/2067; 95% CI: 58.98–63.18; *P* < 0.0001; *χ*^2^ = 219.97, *df* = 1) than in lambs (26.42%; 154/583; 95% CI: 22.9–30.14). Doubtful results were obtained in 3.77% (95% CI: 3.03–4.68) of the adults and 7.38% (95% CI: 5.52–9.79) of the lambs. The highest seroprevalence in adult sheep was observed in western Romania (74.94%; 296/395; 95% CI: 70.44–78.96; *P* < 0.001), while the lowest value was obtained in central Romania (55.82%; 235/421; 95% CI: 51.04–60.49). No sample from adult sheep originated from southeastern Romania. In lambs, the highest seroprevalence was registered in western Romania (48.6%; 52/107; 95% CI: 38.3–58.5; *P* < 0.001), while the lowest value was observed in southeastern Romania (10%; 5/10; 95% CI: 3.3–21.8) (Table 1).

In lambs, the highest seroprevalence of anti-*T. gondii* antibodies was obtained in the 4-month age group (61/132; 46.2%; 95% CI: 37.5–55.1; *P* < 0.001; *χ*^2^ = 276.567, *df* = 9), while in the 2-month category no samples were positive (0/40; 95% CI: 0.0–8.8%) (Table 2).

**Table 2:** *Toxoplasma gondii* seroprevalence in lambs by age by ELISA

Age (months)	*n*	Positive (*n*)	Prevalence (95% CI)	*P*-value
2	40	0	0 (0.0–8.8)	0.001
3	256	84	32.8 (27.1–38.9)	
4	132	61	46.2 (37.5–55.1)*	
5	40	2	5 (0.6–16.9)	
6	99	2	2 (0.2–7.1)	
8	16	5	31.3 (11.0–58.7)	
Total	583	154	26.4 (22.9–30.2)	

*P < 0.05; *n* = number of samples; CI = confidence interval

### Bioassay study

**Fig. 2:**
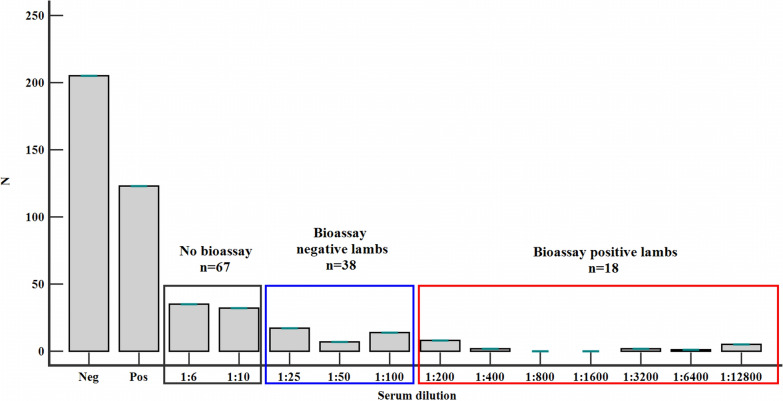
Number of lambs by anti-*Toxoplasma gondii* IgG titer in MAT and positive in bioassay

The seroprevalence of *T. gondii* in lambs sampled for bioassay by MAT was 37.5% (123/328; CI 95% 32.3–43.0) (Table 3), with antibody titers ranging from 1:6 to 1:12,800 (Fig. 2). The titers obtained at serial dilutions in positive samples were: 1:6 in 35 samples; 1:10 in 32 samples; 1:25 in 17 samples; 1:50 in 7 samples; 1:100 in 14 samples; 1:200 in 8 samples; 1:400 in 2 samples; 1:3200 in 2 samples; 1:6400 in 1 sample; 1:12,800 in 5 samples, respectively (Fig. 2).

**Table 3:** Seroprevalence of *T. gondii* infection in lambs by MAT and number of *T. gondii* isolates

Region	MAT				Bioassay (MAT ≥ 1:25)		
	*n*	Positive (*n*)	Prevalence (95% CI)	*P* value	*n*	Positive (*n*)	Genotype
Central	140	40	28.57% (21.74–36.55)	0.001	26	6	–
Northwest	78	56	71.79% (60.97–80.57)*		15	10	II
West	60	17	28.3% (17.5–41.4)		9	0	–
Southeast	50	10	20% (10.0–33.7)		6	2	–
Total	328	123	37.5% (32.3–43.0)		56	18	II

*P < 0.05; *n* = number of samples; CI = confidence interval

Fifty-six out of 123 seropositive lamb sera at MAT had positive results with a titer ≥ 1:25 (Fig. 2). Each of those animals was bioassayed in mice, and viable *T. gondii* was isolated from 18 lambs (32.14%). The isolates obtained on mice originated from lambs sampled in central Romania: Harghita County (*n* = 2), Covasna County (*n* = 2) and Mureș County (*n* = 2), in northwest Romania: Cluj County (*n* = 6) and Maramureș County (*n* = 4) and in southeast Romania: Buzău County (*n* = 2) (Table 3). PCRs performed on DNA extracted from mouse brains confirmed the microscopic results. The bioassay-positive lambs had MAT titer ≥ 1:200.

**Fig. 3:**
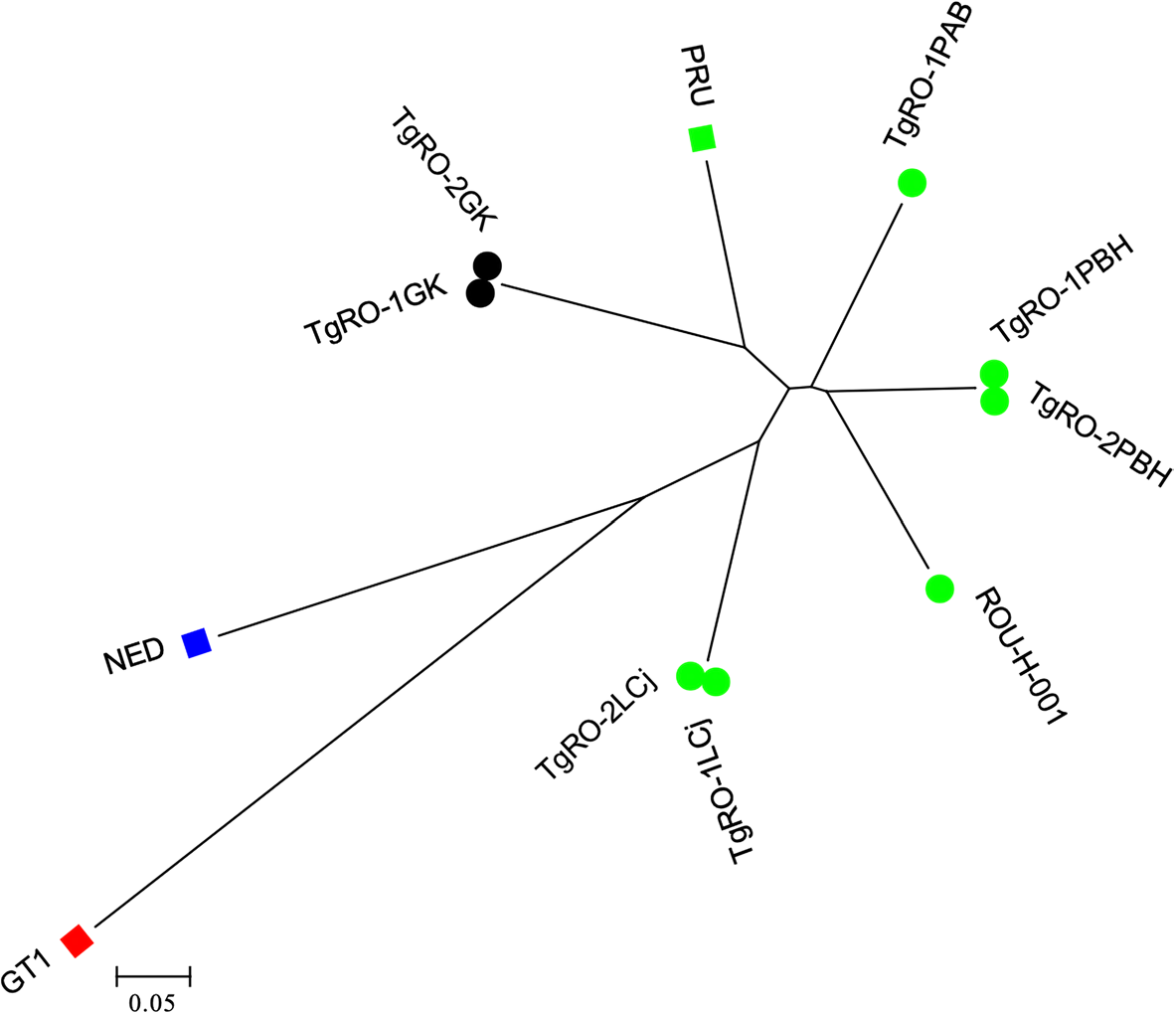
Neighbor-joining tree of *T. gondii* strains from Romania, inferred from calculated Cavalli-Sforza distances for 15 microsatellite markers. The circles indicate the Romanian strains (*n* = 8) with two black circles corresponding to the two strains of this study presenting a complete genotype; the squares correspond to the reference strains (type I in red, type II in green and type III in blue)

Eleven of 18 strains were subjected to genotyping. Only two *T. gondii* strains were genotyped on all 15 microsatellite markers (TgRO-1LCj; TgRO-2LCj). The other four strains (TgRO-3LCj; TgRO-4LCj; TgRO-5LCj; TgRO-6LCj) were incomplete with between 11 and 14 obtained in microsatellite markers. The amount of DNA for the other five strains was inadequate for microsatellite genotyping. *Toxoplasma gondii* isolates shared the same type II genotype with the exception of the missing markers for the four incomplete strains (Table 4, Fig. 3).

**Table 4:** Genotypes of *T. gondii* isolates from sheep, pigs, goats and humans used in our study

Genotype	Isolate ID	Origin	Host species	Microsatellite markers															
				TUB2	W35	TgM-A	B18	B17	M33	IV.1	XI.1	M48	M102	N60	N82	AA	N61	N83	References
II	TgRO-1LCj	Romania	Lamb	289	242	207	158	336	169	274	356	221	174	138	111	277	91	312	This study
II	TgRO-2LCj	Romania	Lamb	289	242	207	158	336	169	274	356	221	174	138	111	277	91	312	This study
II (11/15)	TgRO-3LCj	Romania	Lamb	289	NA	207	158	NA	169	274	356	221	174	138	111	277	NA	NA	This study
II (14/15)	TgRO-4LCj	Romania	Lamb	289	242	207	158	336	169	274	356	NA	174	138	111	277	91	312	This study
II (13/15)	TgRO-5LCj	Romania	Lamb	289	242	207	158	336	169	274	NA	221	174	NA	NA	277	91	312	This study
II (14/15)	TgRO-6LCj	Romania	Lamb	289	242	207	158	336	169	NA	356	221	174	138	111	277	91	312	This study
II	TgRO-1PAB	Romania	Pig	289	242	207	158	336	169	274	356	225	176	142	113	259	95	312	[18]
II	TgRO-1PBH	Romania	Pig	289	242	207	158	336	169	274	356	217	176	142	125	277	93	312	[18]
II	TgRO-2PBH	Romania	Pig	289	242	207	158	336	169	274	356	217	176	142	125	277	93	312	[18]
II	ROU-H-001	Romania	Human	289	242	207	158	336	169	274	356	231	176	138	109	273	93	312	[56]
II	TgRO-1GK	Romania	Goat	289	242	207	158	336	169	274	356	235	176	140	115	275	115	310	[57]
II	TgRO-2GK	Romania	Goat	289	242	207	158	336	169	274	356	235	176	140	115	275	115	310	[57]
Reference strains*																			
I	GT1	SUA	Sheep	291	248	209	160	342	169	274	358	209	168	145	119	265	87	306	[58]
II	PRU	France	Human	289	242	207	158	336	169	274	356	209	176	142	117	265	123	310	[59]
III	NED	France	Human	289	242	205	160	336	165	278	356	209	190	147	111	267	91	312	[60]

*The reference strains are available at the Toxoplasma Biological Resource Center (http://www.toxocrb.com)

The TgRO-1LCj strain has been used previously as a reference strain under the name FR-OVI-ARI061 [56, 57, 61, 62]. In these different studies this strain has been misrepresented as having a French origin, which is not the case. We confirm here that this strain was indeed isolated in the framework of this study

### *Toxoplasma gondii* prevalence in aborted fetuses

*Toxoplasma gondii* DNA was detected in nine (11.84%; 95% CI: 5.56–21.29) of 76 tested aborted/dead fetuses. Both brain and heart samples collected from all the nine aborted fetuses were positive at PCR targeting the 529-bp repeat region. The positive samples belonged to aborted fetuses expelled in the first periods of gestation, and none of the fetuses born dead had presented *T. gondii* DNA.

## Discussion

*Toxoplasma gondii* is responsible for toxoplasmosis, an important zoonotic parasitic diseases, with the potential for severe consequences in not only humans but also all intermediate hosts such as sheep.

The seroprevalence of toxoplasmosis in sheep varies between studies, countries and geographic areas. The seroprevalence of anti-*T. gondii* antibody IgG types in sheep from different European countries is variable: 89% in France [22], 87.4% in Belgium [23], 74% in Great Britain [24], 46.5% in Spain [25], 28% in Italy [26], 27.8% in The Netherlands [27] and 17.2% in Latvia [28]. Other factors that may lead to variations in the seroprevalence of *T. gondii* infection may be the serological tests used, the number of animals tested, the age of the animals and management system [12, 29]. The presence of cats, a history of abortions in sheep in the last 12 months, breed, farm size, semi-intensive growing system, mineral salt supplementation and water origin are considered the risk factors for *T. gondii* infection in sheep [30, 31].

The results of the present study showed that *T. gondii* infection in sheep is widespread in Romania (53.5%). Of the 2650 sera tested, 4.6% had a doubtful result on the ELISA immunoassay technique. Generally, after infection of sheep with *T. gondii* oocysts, IgG antibodies reach the maximum level after 2 months and persist at high titers for several months [1, 32], which may explain some of these doubtful cases. The doubtful reaction can also be due to the common antigens at the level of surface proteins present in some parasites from the phylum Apicomplexa such as *Neospora* spp., *Toxoplasma* spp. and *Hammondia* spp., etc. [33].

Seropositivity in sheep increased in our study with age, adult sheep being exposed to *T. gondii* oocysts for a longer period than lambs [34]. In Romania, previous studies reported a seroprevalence of *T. gondii* antibodies in lambs and adult sheep by ELISA, ranging between 6.5 and 30.6% [35, 36] and 36.3–62.99%, respectively [36, 37]. Our study confirms that age is a risk factor for *T. gondii* infection, the seroprevalence obtained in adults (61.1%) being more than twice that in lambs (26.4%). We investigated lambs aged between 2 and 8 months. No 2-month-old lamb was *T. gondii* seropositive. The highest seroprevalence of anti-*T. gondii* antibodies were obtained in the 4-month group (46.2%). Then, the seroprevalence decreased in the 5- and 6-month-old groups and increased in the 8-month-old group. In ruminants, unlike primates, maternal IgG antibodies are transmitted through colostrum and not transplacentally, and they pass in the bloodstream 24–48 h after birth [38]. Moreover, maternal antibodies decline by 3 months of age, and they disappear by 5 months of age [34]. High antibody titer and age of lambs > 5 months old suggest an immune response caused by an active *T. gondii* infection and not transcolostrally acquired antibodies [34].

Furthermore, a significant statistical difference was noticed in the seroprevalence obtained between the different regions of our study. The differences between regions are probably due to variety in the degree of feline presence according to area and also to climatic conditions, which may influence the sporulation and resistance of *T. gondii* oocysts. Sheep included in the study are grazed from spring to autumn; therefore, cats and wild felids may be an important source of *T. gondii* contamination. Romania has a temperate continental climate, being semi-arid in the southeast of the country and more humid in the regions inside the Carpathian Arc. This may explain the lower seroprevalence in the southeast of Romania. In Serbia, Klun et al. [39] found that region and farm type were associated with *T. gondii* seroprevalence.

The MAT (modified agglutination test) is a serological technique used for the detection of anti-*T. gondii* antibodies in sheep [40, 41]. We have isolated 18 *T. gondii* strains from lambs aged between 2.5 and 4 months with MAT titers ≥ 1:200. Dubey et al. [40] and Dumètre et al. [42] have obtained viable *T. gondii* strains from lambs aged between 6 and 12 months and ewes with MAT titers ≥ 1:50. Therefore, 53 *T. gondii* isolates of 68 lambs' myocardia (77.9%) and 8 *T. gondii* isolates out of 30 ewes' myocardia (26.6%) were obtained in the USA and France, respectively [40, 42]. Likewise, in Spain, from ewe myocardial tissue, 20 (40%) *T. gondii* isolates were obtained out of 50 isolation attempts [43]. The difference between the rates of isolation of the parasites can be explained by the age of tested animals (lambs or ewes), the tissue used for bioassay (diaphragmatic tissue or myocardium), the quantity of tissue used for bioassay (5–25 g diaphragmatic tissue or 50 g myocardium) and the number of mice use for bioassay (2, 5 or 10) [40]. Moreover, a significant correlation was observed between the MAT titer and the percentage of *T. gondii* isolates obtained. If the MAT titer is > 1:16, the probability of obtaining isolates is about 65%. The probability decreases significantly when the MAT titer is < 1:16 [41].

In the present study, 18 strains could be isolated. Six of these 18 isolates could be genotyped using microsatellite markers, obtaining two complete genotypes (TgRO-1LCj; TgRO-2LCj), and four others incomplete with between 11 and 14 markers obtained (TgRO-3LCj; TgRO-4LCj; TgRO-5LCj; TgRO-6LCj). These six genotypes were identical on all markers obtained and belonged to the type 2 lineage. Considering that all six strains came from lambs reared in the same flock from Cluj County, it is probably the same epidemic clone. To our knowledge, this is the first study in Romania to report the isolation and characterization of viable *T. gondii* from seropositive lambs reared in an extensive growth system.

The genetic characterization of *T. gondii* strain circulating in Europe has shown that type II is by far most frequently found in livestock, followed by type III and type I [22, 43–45].

The techniques used for diagnosis of *T. gondii* in sheep abortions can be serological, histological, molecular and biological. *Toxoplasma gondii* genomic DNA detection in various sheep abortion tissues is considered useful and demonstrates congenital transmission of the parasite [46, 47]. Congenital infections occur only when the sheep becomes infected during the pregnancy. To associate *T. gondii* with reproductive disorders, it is necessary to identify the *T. gondii*-like lesions by histological examination and not only to detect the DNA of *T. gondii* [12]. Embryonic death, fetus absorption, fetal death, mummification, abortion and stillbirth are the consequences of the development of macroscopic lesions of the placenta, caused by focal inflammation and necrosis of the cotyledons [48, 49]. However, the isolation of the parasite by bioassay in mice is the “gold standard” in the diagnosis of toxoplasmosis in sheep abortions [50, 51]. The bioassay in mice also has disadvantages, such as ethical aspects and logistical and cost difficulties. Furthermore, its implementation in the routine diagnosis of congenital diseases in sheep is complicated.

In a recent meta-analysis that included global studies on *T. gondii* infection in sheep abortions, a prevalence of 42% by PCR and 16% by serology was reported [52]. The presence of *T. gondii* DNA in sheep fetuses was reported in various studies from Europe, with prevalence ranging between 5.4% in Spain (4/74) [53] and 53.7% in UK [54]. In the present study, 9 (11.8%) out of 76 tested aborted sheep fetuses were found positive by PCR, the DNA being detected in both brain and heart samples. In Spain, Fernández-Escobar et al. [43] found *T. gondii* DNA in 60.3% of fetal brain tissues, placenta tissue and brains from dead weak lambs, originating from 22 abortion outbreaks. However, the prevalence of parasite DNA was different depending on the type of tissue tested: 63.2% in fetal brains, 69.0% in placenta and 11.1% in brains of weak lambs, respectively [43]. Chessa et al. [55] also reported a variable prevalence of *T. gondii* DNA, with values of 3.5% in placenta, 87% in brain and 66.6% in liver samples from sheep fetuses.

## Conclusions

*Toxoplasma gondii* remains a challenge for both human and veterinary medicine. All the knowledge related to the parasite biology, its genetic diversity, risk factors, immune response and epidemiology should be used to reduce the contamination of the environment as well as the transmission of the parasite. The present study reports a high prevalence of *T. gondii* infection in sheep and lambs in Romania and highlights therefore that human contamination by consumption of raw or undercooked sheep/lamb meat is an important risk factor. The study also emphasizes that the role of *T. gondii* as etiological agent associated with abortion and stillbirth in sheep should not be forgotten.

## Data Availability

The data supporting the results and conclusions of this article are included within the article.

## Abbreviations

CI: Confidence interval
ELISA: Enzyme‐linked immunosorbent assay
6‐FAM: 6‐Carboxyfluorescein
HEX: Hexachlorofluorescein
MAT: Modified agglutination test
NED: 2,7′,8′‐Benzo‐5′‐fluoro‐2′,4,7‐trichloro‐5‐carboxyfluorescein
PCR: Polymerase chain reaction
